# Treatment and pathogenesis of acute hyperkalemia

**DOI:** 10.3402/jchimp.v1i4.7372

**Published:** 2012-01-26

**Authors:** Yelena Mushiyakh, Harsh Dangaria, Shahbaz Qavi, Noorjahan Ali, John Pannone, David Tompkins

**Affiliations:** 1 Department of Internal Medicine, Lutheran Medical Center, Brooklyn, NY, USA; 2 St. George's University School of Medicine, Grenada, West Indies

**Keywords:** hyperkalemia, review, treatment, potassium, hyperkalemic

## Abstract

This article focuses on the pathogenesis, clinical manifestations, and various treatment modalities for acute hyperkalemia and presents a systematic approach to selecting a treatment strategy. Hyperkalemia, a life-threatening condition caused by extracellular potassium shift or decreased renal potassium excretion, usually presents with non-specific symptoms. Early recognition of moderate to severe hyperkalemia is vital in preventing fatal cardiac arrhythmias and muscle paralysis. Management of hyperkalemia includes the elimination of reversible causes (diet, medications), rapidly acting therapies that shift potassium into cells and block the cardiac membrane effects of hyperkalemia, and measures to facilitate removal of potassium from the body (saline diuresis, oral binding resins, and hemodialysis). Hyperkalemia with potassium level more than 6.5 mEq/L or EKG changes is a medical emergency and should be treated accordingly. Treatment should be started with calcium gluconate to stabilize cardiomyocyte membranes, followed by insulin injection, and b-agonists administration. Hemodialysis remains the most reliable method to remove potassium from the body and should be used in cases refractory to medical treatment. Prompt detection and proper treatment are crucial in preventing lethal outcomes.

Severe hyperkalemia, a potentially life-threatening condition, can cause muscle paralysis and lethal cardiac arrhythmias. It should be treated in a timely manner employing all available resources. A retrospective chart review at our institution of patients treated with cation exchange resin demonstrated inconsistencies in the management of hyperkalemia. In 71% of patients, a cation exchange resin was administered, without appropriate indications, without alternative measures being employed, or when contraindicated. These findings are probably not unique to our institution and thus support the need for a more systematic approach to the assessment and management of hyperkalemia.

This article focuses on the pathogenesis of hyperkalemia, its clinical manifestations, and various treatment modalities for acute hyperkalemia. We hope to educate clinicians and house staff about the indications and methods of treatment of hyperkalemia so that they will develop a systematic approach and integrate all aspects of the hyperkalemic patient's history and current condition when selecting their treatment strategy.

## Findings of retrospective study

A randomized, retrospective chart review of 65 medical records from patients who received Kayexalate between November 2007 and November 2008 was conducted. Data were collected and analyzed for the following outcomes: Kayexalate administered without following proper indication or when contraindicated, administration resulting in serum electrolyte abnormalities, and other adverse effects within 12 hours of administration. Forty-one females and 24 males from the medicine, surgery, and obstetrics and gynecology departments were reviewed in this study and analysis of the data revealed the following values: Kayexalate was administered without following proper indications (defined as moderate to severe hyperkalemia), with absolute contraindications, or with drug contraindications; and no alternative modalities were employed in 46 (71%) of the patients. Electrolyte disturbances pretreatment were noted to be as follows: hypocalcemia in 9% of the patients, hypomagnesemia in 0% of the patients, and hypernatremia in 9% of the patients. Absolute medical contraindications were noted in 6% of the patients sampled. Relative medical contraindications were noted in 88% of the patients and drug contraindications were noted in 37% of the patients receiving Kayexalate. In the 17 patients with posttreatment electrolyte disturbances or adverse effects, 13 (77%) of them were given Kayexalate when contraindicated or unindicated, with no alternative modalities employed. The posttreatment electrolyte disturbances were as follows: hypocalcemia in 15% of the patients, hypomagnesemia in 3% of the patients, hypernatremia in 11% of the patients, and hypokalemia in 2%. In the first 12 hours after treatment, 6% of patients developed adverse effects. The appropriate dosage of the medication was administered in 100% of the patients.

### Pathogenesis of hyperkalemia

The basic pathophysiology of hyperkalemic states involves either extracellular potassium shifts or decreased renal excretion. Common etiologies leading to measurement of hyperkalemia include pseudohyperkalemia, decreased renal excretion, and abnormal potassium distribution. Increased dietary potassium intake or other exogenous sources rarely cause more than transient hyperkalemic states unless underlying pathology is present. Similarly, during increased potassium release from endogenous sources, such as high cell turnover or tissue damage, hyperkalemic states are transient, unless concomitant renal pathology is present. Chronic hyperkalemia is always associated with renal potassium excretion defects. It should be noted that frequently multiple etiologies present simultaneously and may obscure the picture.

### Pseudohyperkalemia (fictitious hyperkalemia)

Pseudohyperkalemia commonly arises from shifts of potassium from blood cells to blood plasma by mechanical trauma during venipuncture or during the clotting process *in vitro*. These effects are further enhanced when there is marked leukocytosis or thrombocytosis. A rare form of pseudohyperkalemia, familial pseudohyperkalemia, causes potassium to leak out of excessively permeable erythrocyte membranes *in vitro*. *In vivo*, however, this disorder does not contribute to hyperkalemia because the leaked potassium is renally excreted (1, 2).

### Decreased renal excretion

The kidney has a central role in normal potassium homeostasis with the distal components of the nephron responsible for the bulk of potassium excretion. The renal abnormalities that manifest in hyperkalemia can be grouped as follows: renal tubular secretory abnormalities, impaired renin-aldosterone axis, drug-induced hyperkalemia, decreased distal tubular flow with low sodium, and renal failure.

#### Renal tubular secretory abnormalities

Common renal tubular secretory abnormalities that can lead to hyperkalemia are type 1 (distal) renal tubular acidosis, renal disease in sickle cell disease and systemic lupus erythematosus, renal transplant, and obstructive uropathy.

#### Impaired renin-aldosterone axis

Impaired renin-aldosterone axis can cause enhanced hyperkalemia. This occurs in Addison's disease, adrenal enzyme deficiencies (21 hydroxylase, corticosterone methyl oxidase), hyporeninemic hypoaldosteronism, and angiotensin deficiency or insensitivity. Furthermore, medications can impair the renin-aldosterone axis, including prostaglandin inhibitors (indomethacin, ibuprofen, piroxicam, aspirin, naproxen, fenoprofen, and sulindac), beta-adrenergic antagonists, angiotensin-converting enzyme inhibitors (ACEI), angiotensin receptors blockers (ARB), tacrolimus, and heparin.

#### Drug-induced hyperkalemia

In addition to interfering with the renin-aldosterone axis, medications can cause hyperkalemia by other mechanisms. Potassium-sparing diuretics (amiloride and triamterene), trimethoprim, and pentamidine all block sodium reabsorption in the distal nephron, reducing the luminal voltage gradient, and decreasing potassium excretion rates. Spironolactone blocks aldosterone receptors, and cyclosporine causes hyperkalemia by enhancing chloride reabsorption.

#### Decreased distal tubular flow with low sodium

A significant decrease in sodium delivery and/or tubular flow rate at the distal nephron also causes hyperkalemia. These are commonly seen in patients with either underlying renal disease or Addison's disease, who may develop acute pulmonary edema or have intravascular volume depletion.

#### Renal failure

Acute tubular necrosis and interstitial nephritis are the common causes of oliguric acute kidney failure. The distal tubules and collecting duct cells are often damaged and thus unable to excrete potassium. Additionally, as explained above, the distal delivery of sodium and/or the distal tubular flow rate is often decreased, once again causing hyperkalemia. In chronic kidney disease (CKD), the diminished tubular mass is less tolerant to acute potassium challenges; therefore, these patients are at increased risk for developing hyperkalemia (1, 2).

### Abnormal potassium distribution

Distribution abnormalities of potassium are seen during metabolic acidosis, insulin deficiency, aldosterone deficiency, adrenergic antagonists, and tissue damage. During metabolic acidosis, there is a significant extracellular shift of intracellular potassium in exchange for protons leading to hyperkalemia. Insulin also maintains potassium balance between extracellular and intracellular compartments, and decrease in insulin causes a rise in extracellular potassium (commonly seen in diabetic patients). Furthermore, serum hypertonicity from hyperglycemia enhances hyperkalemia. Hypoaldosteronism, in addition to diminishing renal potassium excretion, causes decreased uptake of potassium by non-renal cells. On the other hand, catecholamines and beta-agonist enhance potassium uptake by cells via the beta-2 adrenergic receptors on cells, and when these receptors are unavailable due to antagonist actions, hyperkalemia occurs. However, administration of propranolol causes out of proportion hyperkalemia due to potassium efflux from muscles combined with its antiadrenergic effects. There can also be a rise in extracellular potassium during significant tissue damage, and if accompanied by acute kidney injury, hyperkalemia will be sustained. Other causes of hyperkalemia from potassium shifts include severe exercise, hyperkalemic periodic paralysis, cardiac surgery, insulin antagonists (somatostatin and diazoxide), hypertonic solutions (hypertonic saline and hypertonic mannitol), digitalis overdose, succinylcholine, arginine hydrochloride, lysine hydrochloride, and fluoride poisoning (1, 2).

When the etiology of hyperkalemia is not apparent, an assessment of renal potassium excretion by measuring urine osmolality and spot potassium levels to determine the transtubular potassium gradient [TTKG=(urine K/serum K)/(Urine osmolality/Serum osmolality) can help clarify the cause. Hyperkalemia with a TTKG of >7 suggests normal aldosterone function and that renal tubular mechanisms for potassium excretion are intact. This may be seen in the setting of decreased filtrate delivery to the distal nephron with volume depletion. A TTKG <7 suggests impaired potassium secretion secondary to hypoaldosteronism. Measurement of the serum aldosterone level will distinguish adrenal disease or hyporeninemic hypoaldosteronism from aldosterone resistance.

### Clinical manifestations of hyperkalemia

Clinical manifestations of mild to moderate hyperkalemia are usually non-specific and may include generalized weakness, fatigue, nausea, vomiting, intestinal colic, and diarrhea. Severe hyperkalemia may lead to life-threatening conditions such as cardiac arrhythmias and muscle paralysis.

Potassium and sodium play a key role in the function of the myocardium; therefore, their concentration gradients are strictly maintained. Any imbalance of this concentration gradient affects the ability of the heart to maintain a normal rhythm. The concentration gradient is maintained by the sodium potassium ATPase pumps located on the cellular membrane that actively pump sodium outside and potassium inside the cell. When the potassium level increases in the extracellular space, the potassium concentration gradient across the cellular wall decreases; and this decreases the resting membrane potential. The change in resting membrane potential caused by hyperkalemia is the principle pathophysiologic mechanism behind most of its symptoms. The decrease in the resting membrane potential decreases the number of sodium channels activated that in turn decrease the magnitude of inward sodium current. This causes a prolonged conduction of the impulse with prolonged depolarization (3).

As the myocardium is highly sensitive to any changes in potassium ion concentration, the imbalance of the potassium concentration gradient in hyperkalemia can cause a progression of EKG changes such as increased T wave amplitude (peaked T waves), prolongation of the PR interval and QRS duration, loss of P waves, AV conduction delay, culminating in the merging of the QRS complex with the T wave producing a sine wave pattern, and asystole (3, 4). Clinically, patients can present with palpatations, syncope, and sudden cardiac death.

Furthermore, hyperkalemia causes sustained spontaneous depolarization of skeletal muscles that leads to inactivation of sodium channels of the muscle membrane. These changes can produce the symptoms of muscle weakness and in extreme cases, paralysis (5, 6).

### Treatment of hyperkalemia

Treatment of hyperkalemia can be divided into acute and long-term therapy. The approach to treatment differs in patients with acute renal failure and CKD. In patients with chronic renal disease, a new steady state develops in which potassium excretion is stimulated at a different, higher, extracellular potassium level, so that it again matches the intake. When this new steady state is reached, plasma potassium remains stable unless a new event occurs that shatters the balance (7). A recent retrospective study of over 245,000 patients examined the frequency of hyperkalemia and the impact of renal dysfunction (8). For each stage of renal function (normal to Stage 5 CKD) as the level of hyperkalemia increased, the odds of death increased. However, for a given level of hyperkalemia, there was an inverse relationship between the stage of CKD and odds of 1-day mortality after a hyperkalemic event (8).

**Fig. 1:**
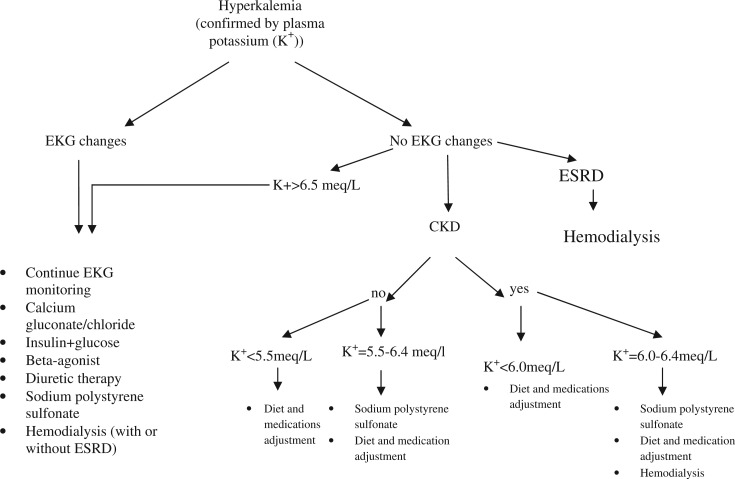
Guide to the treatment of hyperkalemia.

Treatment of acute hyperkalemia, regardless of its causes, depends on potassium level, and presence or absence of EKG changes (Fig. 1). Emergent treatment should be administered if EKG changes are present or if plasma potassium level is more than 6.5 mEq/L regardless of the EKG changes (Table 1). The goal of acute therapy is to stabilize the cardiomyocyte membranes to prevent arrhythmia, shift potassium into the cells, and enhance elimination of potassium from the body.

**Table 1:** Emergency management of acute hyperkalemia^a^

Medication	Response type	Onset of action	Duration of action	Mechanism of action	Expected decrease in potassium level
Calcium gluconate	rapid	1–2 min	30–60 min	Protect cardiomyocytes	0.5–1.5 mEq/L
Glucose + insulin	intermediate	10–20min	2–6 hours	Shift potassium intracellularly	0.5–1.5 mEq/L (dose dependent)
Beta-agonists	intermediate	3–5min	1–4 hours	Shift potassium intracellularly	
Sodium bicarbonate (only in patients with metabolic acidosis, bicarbonate < 22mEq/L)	intermediate	30–60min	2–6 hours	Shift potassium intracellularly (questionable effect)	
Exchange resin	delayed	2–6hours	4–6 hours	Elimination of potassium from the body	
Furosemide	delayed	5–30 min	2–6 hours	Elimination of potassium from the body	
Hemodialysis	delayed	immediate		Elimination of potassium from the body	1mmol/L in the first 60 min and total of 2mmol/l by 180 min

a Synthesized from Sood et al. (9), Weisberg (10), Mandelberg et al. (11), Zender et al. (12), Khanna et al. (13), and Pancu et al. (14).

Long-term therapy of hyperkalemia includes diet and medication modifications. A low potassium diet should be discussed with the patient, once the acute issues are resolved. Medications should be reevaluated with special attention paid to ACEI, ARB, and potassium-sparing diuretics.

## Cardiac stabilization

### Calcium

Calcium salts antagonize the effects of potassium on cardiomyocyte membranes without affecting plasma potassium level. In EKG, if abnormalities are present or the plasma potassium level is greater than 6.5 mEq/L, calcium therapy is indicated to help prevent the development of potential lethal arrhythmias, while other measures to lower potassium levels are instituted (below). Calcium is usually given as IV injection of 10 cc 10% calcium gluconate over 5–10 min. The patient should be on a cardiac monitor, and EKG may be repeated after calcium administration. If EKG changes persist after 5–10 min, a second injection of calcium should be repeated in 5 min. Calcium should be administered cautiously and monitored closely in patients who take digitalis, especially those with a high level of digoxin in their blood. Hypercalcemia can increase the cardiotoxic effects of digitalis. For added safety, the same dose of calcium (10 cc of 10% calcium gluconate) is added to 100 cc of 5% dextrose in water and infused over 20–30 min to avoid transient hypercalcemia. In the setting of hyperkalemia secondary to digitalis toxicity, the use of digitoxin-specific antibody is indicated.

## Shift potassium into cells

### Insulin and glucose

Insulin is an effective and reliable drug that causes potassium to shift into cells by increasing Na–K-ATPase activity. Serum potassium level starts trending down within 10–20 min of insulin and glucose administration with maximal action in 60 min: The effect lasts for 2–6 hours. Bolus of 10 units of insulin with 25–50 g of glucose should be given as an intravenous injection. Patients with hyperglycemia can be given insulin alone to avoid worsening of hyperkalemia by hyperosmolar state. Blood sugar levels should be closely monitored to avoid hypoglycemia (9).

### Beta-agonists

Beta-adrenergic agonists also activate Na–K-ATPase and cause potassium to shift into cells. The usual dose of albuterol is 10–20 mg via nebulizer – at least four times higher than the usual dose used for patients with bronchospasm.

The most common side effects of beta-agonists are tachycardia and tremors. Inhaled beta-agonists are usually ineffective in patients taking beta-blockers and generally ineffective in 30%–40% of patients for unknown reasons. However, studies showed that the effect of beta-agonists and insulin together is additive in lowering potassium level, superior to using each of them alone, and may prevent insulin-induced hypoglycemia (15, 16).

### Sodium bicarbonate

The use of bicarbonate therapy is controversial in patients without metabolic acidosis. A review paper by Kamel and Wei cited several studies demonstrating that short-term administration of bicarbonate does not reduce potassium level and may cause hypernatremia, hypocalcaemia, metabolic alkalosis, and hypervolemic state (17). In patients with metabolic acidosis (bicarb <22 mEq/L), bicarbonate can be given as a bolus dose of 50 mEq/L or as continuous effusion but the mechanism of action has not been clarified yet (18). To the best of our knowledge, there were no studies that showed the benefits of short-term bicarbonate therapy in the absence of metabolic acidosis.

## Elimination of potassium from the body

### Diuretic therapy

The use of loop diuretics, such as furosemide 40–80 mg IV, in combination with saline infusion to ensure delivery of sodium to the distal nephron can promote renal potassium excretion in patients with normal kidney functions. Chronic diuretic therapy can be used in patients with CKD and mild hyperkalemia.

### Cation-exchange resins

Sodium-polysterene sulfonate (SPS) may be used orally and in the form of retention enema and works primarily in the large intestine by exchanging sodium for potassium ions. The onset of action is unpredictable and usually takes a few hours. SPS can cause constipation, so it is usually given with a cathartic agent, such as sorbitol. The resin is not used as a first-line treatment for hyperkalemia because of its slow onset of action and lack of immediate effect. There are no established guidelines about its dosage in correlation to potassium level, and many institutions have developed their own protocols. A retrospective review of potassium levels in people receiving only resin demonstrated that 30 g of resin resulted in an average decrease in serum potassium of 0.99 mEq/L with a standard deviation of 0.64 (19).

One of the major side effects of SPS in sorbitol is colonic necrosis associated with increased morbidity and mortality. Patients may be susceptible to intestinal ischemia even in the absence of end stage renal disease, surgical intervention, or significant comorbidity (20, 21).

### Hemodialysis

Hemodialysis is the therapy of choice for life-threatening hyperkalemia in patients with compromised renal function, severe rhabdomyolysis, or for severe hyperkalemia that is not responsive to medical management. Plasma potassium drops by 1mmol/L during the first hour of hemodialysis, with a total drop of about 2mmol/L in 3 hours, and then reaches a nadir and remains stable at 4 hours (22). Rebound always occurs after dialysis, and the magnitude of post rebound plasma potassium is proportional to the predialysis potassium level (18). In addition, electrolytes should be closely monitored for at least 24 hours after hemodialysis is administered.

## Conclusion

Moderate to severe hyperkalemia requires immediate treatment and close monitoring to prevent the development of cardiac arrhythmias and muscle paralysis. This article presented guidelines to aid clinicians in their diagnosis and treatment of this potentially life-threatening condition. All patients with confirmed hyperkalemia should be assessed immediately with an EKG to rule out serious cardiac arrhythmias. Calcium gluconate should be used as a first-line agent in patients with EKG changes or severe hyperkalemia to protect cardiomyocytes. Insulin and glucose combination is the fastest acting drug that shifts potassium into the cells. B-agonists can be used in addition to insulin to decrease plasma potassium levels. Sodium bicarbonate is effective only in patients with metabolic acidosis; otherwise, its usage remains controversial. Exchange resin has very slow action and is therefore indicated for treatment of chronic hyperkalemia. Hemodialysis is the most effective and reliable method to remove potassium from the body. Prompt and aggressive treatment of hyperkalemia may help to avoid complications and prevent patient mortality.
